# Effect of AQP Inhibition on Boar Sperm Cryotolerance Depends on the Intrinsic Freezability of the Ejaculate

**DOI:** 10.3390/ijms20246255

**Published:** 2019-12-11

**Authors:** Ariadna Delgado-Bermúdez, Marc Llavanera, Sandra Recuero, Yentel Mateo-Otero, Sergi Bonet, Isabel Barranco, Beatriz Fernandez-Fuertes, Marc Yeste

**Affiliations:** Biotechnology of Animal and Human Reproduction (TechnoSperm), Unit of Cell Biology, Department of Biology, Institute of Food and Agricultural Technology, Faculty of Sciences, University of Girona, E-17003 Girona, Spain; ariadna.delgado@udg.edu (A.D.-B.); marc.llavanera@udg.edu (M.L.); sandra.recuero@udg.edu (S.R.); yentel.mateo@udg.edu (Y.M.-O.); sergi.bonet@udg.edu (S.B.); isabel.barranco@udg.edu (I.B.); beatriz.fernandez@udg.edu (B.F.-F.)

**Keywords:** aquaporins, boar, phloretin, propanediol, spermatozoa

## Abstract

Aquaporins (AQPs) are transmembrane channels with permeability to water and small solutes that can be classified according to their structure and permeability into orthodox AQPs, aquaglyceroporins (GLPs), and superAQPs. In boar spermatozoa, AQPs are related to osmoregulation and play a critical role in maturation and motility activation. In addition, their levels differ between ejaculates with good and poor cryotolerance (GFE and PFE, respectively). The aim of this work was to elucidate whether the involvement of AQPs in the sperm response to cryopreservation relies on the intrinsic freezability of the ejaculate. With this purpose, two different molecules: phloretin (PHL) and 1,3-propanediol (PDO), were used to inhibit sperm AQPs in GFE and PFE. Boar sperm samples were treated with three different concentrations of each inhibitor prior to cryopreservation, and sperm quality and functionality parameters were evaluated in fresh samples and after 30 and 240 min of thawing. Ejaculates were classified as GFE or PFE, according to their post-thaw sperm viability and motility. While the presence of PHL caused a decrease in sperm quality and function compared to the control, samples treated with PDO exhibited better quality and function parameters than the control. In addition, the effects of both inhibitors were more apparent in GFE than in PFE. In conclusion, AQP inhibition has more notable consequences in GFE than in PFE, which can be related to the difference in relative levels of AQPs between these two groups of samples.

## 1. Introduction

The amphipathic nature of the plasma membrane is a limiting factor for simple diffusion of water molecules at high rates. In contrast, proper cell function and homeostasis strictly depend on the permeability of the plasma membrane to water and solutes [1]. Aquaporins (AQPs), a family of transmembrane proteins ubiquitously present in mammalian cells, facilitate the diffusion of water and some small solutes through the plasma membrane [2]. Mammalian AQPs are classified into three different groups according to their sequence similarity and their solute permeability: orthodox AQPs, aquaglyceroporins (GLPs) and superAQPs; that include a total of 13 members (AQP0-AQP12). Orthodox AQPs are exclusively permeable to water and include AQP0, AQP1, AQP2, AQP4, AQP5, AQP6, and AQP8. GLPs include AQP3, AQP7, AQP9, and AQP10, which are permeable to water, glycerol and other small molecules. Finally, AQP11 and AQP12 are members of the superAQPs group, and are involved in the transport of both water and glycerol, presenting deep structural differences with the members of the other groups. Even if ubiquitous, the presence of AQPs differs between species and also between cell types. Concerning mammalian spermatozoa, AQP3, AQP7, and AQP11 have been identified in the pig [3,4], horse [5], mouse [6,7,8], human [9,10], and bull [11,12]; AQP8 has been found in mouse [7] and human spermatozoa [9,10]; and AQP9 is present in pig spermatozoa [13]. The importance of AQPs in spermatozoa relies on their ability to regulate cell volume and osmotic balance, thus having a key role in spermatogenesis [14] and post-ejaculatory events, such as the activation of sperm motility and the adaptation to the female environment (reviewed by [2]).

To date, sperm cryopreservation is the most efficient method for long-term storage of boar spermatozoa [15]. Nevertheless, this procedure is a source of osmotic stress causing damage to the sperm nucleus, intracellular organelles, and plasma membrane, and reducing sperm survival and fertilizing capacity [15,16]. Cryotolerance, which can be defined as the ability of sperm to withstand the detrimental effects of the cryopreservation process, is highly variable between different mammalian species, and it depends, among other factors, on differences in membrane composition [16,17,18,19,20]. Moreover, within a given species, males with similar fresh sperm quality present highly variable cryotolerance, both intra- and inter-individually [21,22,23]. For this reason, ejaculates can be classified as having good (GFE) or poor freezability (PFE), depending on their post-thaw sperm quality and functional parameters, mainly viability and motility [21]. Because the levels of some proteins differ between GFE and PFE, they can be used as biomarkers of cryotolerance in fresh samples [24]. The key role of AQPs in osmoregulation is a potential mechanism through which these proteins might be involved in sperm cryopreservation, which has previously been confirmed in bull [11,12], boar [25,26], and stallion [5], since spermatozoa from GFE and PFE present different levels of some AQPs. In effect, AQP3 and AQP7 are related to the cryotolerance of boar spermatozoa [25], AQP7 [3] and AQP11 [12] are associated with that of bull spermatozoa, and AQP3 and AQP11 are related to stallion sperm freezability [5]. Moreover, inhibition of AQPs alters boar and stallion sperm cryotolerance [26,27].

Different AQP inhibitors have been previously tested in other cell types, such as 1,3-propanediol (PDO) and phloretin (PHL). PDO has been proven to occlude, with high affinity, the pore channel of orthodox AQPs from the outer side of the plasma membrane and with high affinity [28,29]. In addition, PDO is able to penetrate through *Plasmodium falciparum Pf* AQP (which is an analogue of AQP3, AQP7, and AQP9) and to mildly inhibit this GLP analogue from the inner part of the plasma membrane [28]. PHL inhibits AQP3 and AQP9, which are both GLPs [30,31]. Considering the differences on AQP levels between GFE and PFE [25] and the impact of AQP inhibition on sperm cryotolerance [26], it is reasonable to suggest that the effects of AQP inhibition might vary between GFE and PFE. Therefore, this study aims to elucidate whether the sperm response to AQP inhibition (through incubation with PDO and PHL) prior to cryopreservation differs between GFE and PFE.

## 2. Results

### 2.1. Classification of Boar Ejaculates in GFE and PFE Groups

Classification of ejaculate pools by cluster analysis resulted in 11 GFE and 10 PFE. In fresh samples, no differences were identified in any of the parameters measured between groups (*p* > 0.05). However, sperm total and progressive motility, viability, percentage of viable cells with low membrane lipid disorder and percentage of cells with high mitochondrial membrane potential were higher in GFE than in PFE at both 30 min and 240 min post-thaw (*p* < 0.05).

### 2.2. Effects of AQP Inhibition on Cryopreserved Sperm Quality Parameters

**Figure 1:**
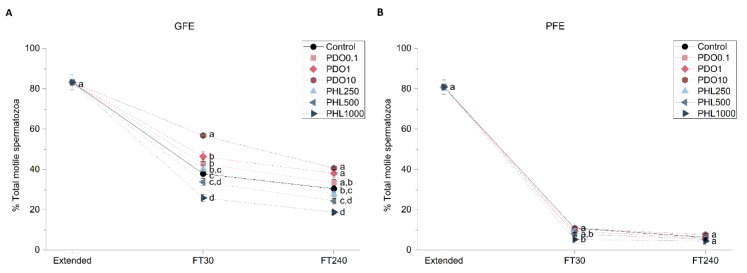
Percentages of total motile spermatozoa (TMOT) in good freezability ejaculates (GFE; (**A**)) and poor freezability ejaculates (PFE; (**B**)) exposed to AQP inhibitors in the extender: 0.1 mmol/L, 1 mmol/L and 10 mmol/L 1,3-propanediol (PHL) or 250 µmol/L, 500 µmol/L and 1000 µmol/L phloretin (PHL); or not (control). Data were collected from fresh (extended) and frozen-thawed (FT) samples at 30 and 240 min post-thaw, and are shown as mean ± SEM. Different letters (a, b, c, d) indicate a significant difference (*p* < 0.05) between treatments within a given time point.

**Figure 2:**
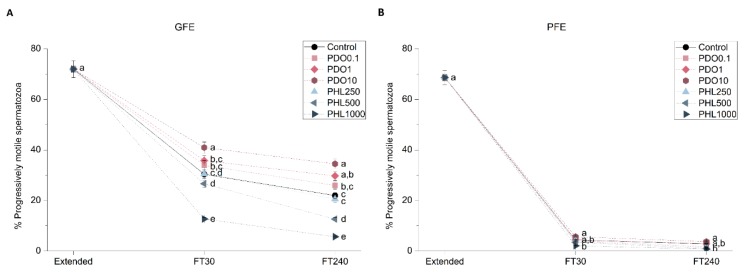
Percentages of progressively motile spermatozoa (PMOT) in good freezability ejaculates (GFE; (**A**)) and poor freezability ejaculates (PFE; (**B**)) exposed to AQP inhibitors in the extender: 0.1 mmol/L, 1 mmol/L, and 10 mmol/L 1,3-propanediol (PHL) or 250 µmol/L, 500 µmol/L, and 1000 µmol/L phloretin (PHL); or not (control). Data were collected from fresh (extended) and frozen-thawed (FT) samples at 30 and 240 min post-thaw, and are shown as mean ± SEM. Different letters (a, b, c, d, e) indicate a significant difference (*p* < 0.05) between treatments within a given time point.

**Figure 3:**
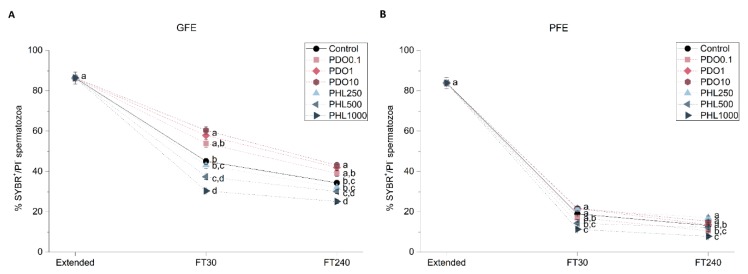
Percentages of SYBR14^+^/PI^−^ spermatozoa in good freezability ejaculates (GFE; (**A**)) and poor freezability ejaculates (PFE; (**B**)) exposed to AQP inhibitors in the extender: 0.1 mmol/L, 1 mmol/L, and 10 mmol/L 1,3-propanediol (PHL) or 250 µmol/L, 500 µmol/L, and 1000 µmol/L phloretin (PHL); or not (control). Data were collected from fresh (extended) and frozen-thawed (FT) samples at 30 and 240 min post-thaw, and are shown as mean ± SEM. Different letters (a, b, c, d) indicate a significant difference (*p* < 0.05) between treatments within a given time point.

**Figure 4:**
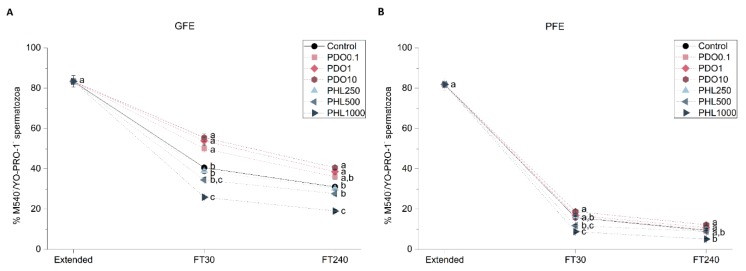
Percentages of viable spermatozoa with low membrane lipid disorder (M540^−^/YO-PRO-1^−^) in good freezability ejaculates (GFE; (**A**)) and poor freezability ejaculates (PFE; (**B**)) exposed to AQP inhibitors in the extender: 0.1 mmol/L, 1 mmol/L, and 10 mmol/L 1,3-propanediol (PHL) or 250 µmol/L, 500 µmol/L, and 1000 µmol/L phloretin (PHL); or not (control). Data were collected from fresh (extended) and frozen-thawed (FT) samples at 30 and 240 min post-thaw, and are shown as mean ± SEM. Different letters (a, b, c) indicate a significant difference (*p* < 0.05) between treatments within a given time point.

**Figure 5:**
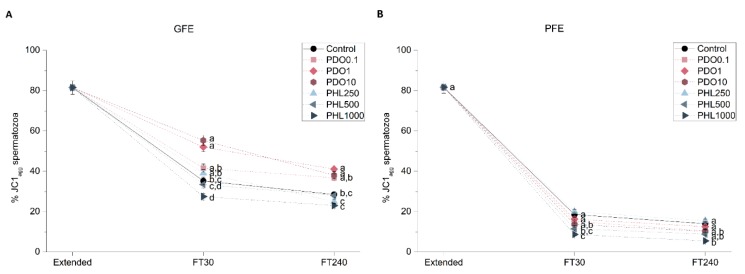
Percentages of spermatozoa with high mitochondrial membrane potential (JC1_agg_) in good freezability ejaculates (GFE; (**A**)) and poor freezability ejaculates (PFE; (**B**)) exposed to AQP inhibitors in the extender: 0.1 mmol/L, 1 mmol/L, and 10 mmol/L 1,3-propanediol (PHL) or 250 µmol/L, 500 µmol/L, and 1000 µmol/L phloretin (PHL); or not (control). Data were collected from fresh (extended) and frozen-thawed (FT) samples at 30 and 240 min post-thaw, and are shown as mean ± SEM. Different letters (a, b, c, d) indicate a significant difference (*p* < 0.05) between treatments within a given time point.

**Figure 6:**
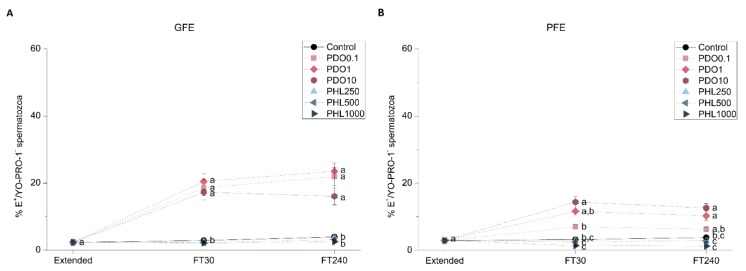
Percentages of viable spermatozoa with high levels of superoxides (E^+^/YO-PRO-1^−^) in good freezability ejaculates (GFE; (**A**)) and poor freezability ejaculates (PFE; (**B**)) exposed to AQP inhibitors in the extender: 0.1 mmol/L, 1 mmol/L, and 10 mmol/L 1,3-propanediol (PHL) or 250 µmol/L, 500 µmol/L, and 1000 µmol/L phloretin (PHL); or not (control). Data were collected from fresh (extended) and frozen-thawed (FT) samples at 30 and 240 min post-thaw, and are shown as mean ± SEM. Different letters (a, b, c) indicate a significant difference (*p* < 0.05) between treatments within a given time point.

**Figure 7:**
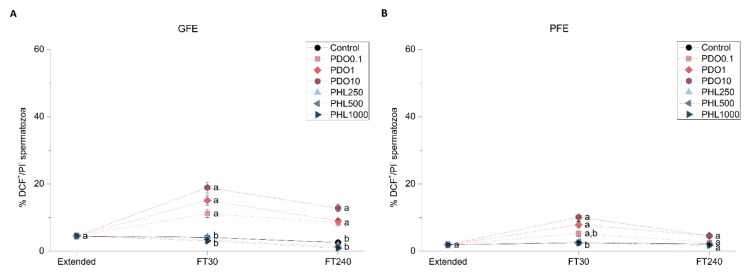
Percentages of viable spermatozoa with high levels of peroxides (DCF^+^/PI^−^) in good freezability ejaculates (GFE; (**A**)) and poor freezability ejaculates (PFE; (**B**)) exposed to AQP inhibitors in the extender: 0.1 mmol/L, 1 mmol/L, and 10 mmol/L 1,3-propanediol (PHL) or 250 µmol/L, 500 µmol/L, and 1000 µmol/L phloretin (PHL); or not (control). Data were collected from fresh (extended) and frozen-thawed (FT) samples at 30 and 240 min post-thaw, and are shown as mean ± SEM. Different letters (a, b) indicate a significant difference (*p* < 0.05) between treatments within a given time point.

To determine the effects of AQP inhibitors during cryopreservation, quality and function parameters of both fresh and frozen-thawed spermatozoa from GFE and PFE samples were evaluated. No differences were observed in fresh samples for any parameter measured between treatments and the control (*p* > 0.05), since inhibitors were added immediately before cryopreservation (Figure 1, Figure 2, Figure 3, Figure 4, Figure 5, Figure 6 and Figure 7). After thawing, samples from the PFE group presented significantly (*p* < 0.05) lower values of all sperm quality and function parameters than GFE in the presence of all treatments and in the control group.

#### 2.2.1. Sperm Motility

Figure 1 and Figure 2 show the percentage of total motility (TMOT, %) and progressive motility (PMOT, %) in fresh and frozen-thawed samples exposed to different AQP inhibitors during cryopreservation. In GFE, TMOT was higher when samples were treated with PDO than in the control (*p* < 0.05), whereas in PFE there were no differences with regard to the control when exposed to different PDO concentrations (*p* > 0.05). On the other hand, in the presence of 1000 µmol/L PHL, TMOT was lower than in the control at 30 min post-thaw (*p* < 0.05) in both GFE and PFE, whereas, at 240 min post-thaw, this difference was only observed in GFE (*p* < 0.05). At 30 min post-thaw, treatment with 10 mmol/L PDO caused an increase in PMOT, whereas at 240 min this change was observed at both 1 and 10 mmol/L concentrations in GFE (*p* < 0.05). Concerning samples treated with PHL, PMOT was lower than in the control at the 1000 µmol/L concentration at 30 min post-thaw, whereas at 240 min post-thaw this effect was observed at both 500 and 1000 µmol/L concentrations in GFE (*p* < 0.05). PMOT was not affected by any inhibitor in PFE (*p* > 0.05).

#### 2.2.2. Sperm Viability

Sperm viability was assessed with the SYBR14/propidium iodide (PI) test in order to determine the effects of different concentrations of the inhibitors during cryopreservation (Figure 3). Sperm viability was higher in GFE treated with 1 mmol/L and 10 mmol/L PDO at both 30 and 240 min post-thaw (*p* < 0.05). However, PDO had no effect in sperm viability in PFE, as evidenced by a lack of differences between PDO-exposed sperm samples and the control (*p* > 0.05). Concerning the PHL treatments, sperm viability in both GFE and PFE was lower in the presence of both 500 and 1000 µmol/L PHL at 30 min post-thaw (*p* < 0.05). Nevertheless, at 240 min post-thaw, sperm viability was only lower than the control in the presence of the highest concentration of PHL (*p* < 0.05).

#### 2.2.3. Sperm Membrane Lipid Disorder

Sperm membrane lipid disorder was evaluated through the merocyanine 540 and YO-PRO-1 co-staining test (Figure 4). When PDO was added to GFE samples, there was an increase in the percentage of spermatozoa with low membrane lipid disorder at 30 min post-thaw (*p* < 0.05), whereas this effect did not persist at 240 min post-thaw in the 0.1 mmol/L group (*p* > 0.05). No effect was observed when PDO was added to PFE samples (*p* > 0.05). Addition of 1000 μmol/L PHL to GFE samples caused a decrease in the percentage of spermatozoa with low membrane lipid disorder at both 30 and 240 min post-thaw (*p* < 0.05). However, in PFE samples, this same PHL concentration caused a decrease at 30 min after-thaw (*p* < 0.05), which was not observed at 240 min post-thaw (*p* > 0.05).

#### 2.2.4. Mitochondrial Membrane Potential (MMP)

Mitochondrial membrane potential (MMP) was evaluated through JC1-staining to determine the effects of AQP inhibition on GFE and PFE (Figure 5). The addition of PDO to GFE samples caused an increase in the percentage of spermatozoa with high MMP (spermatozoa with JC1 aggregates, JC1_agg_) at the highest concentrations, at both 30 min and 240 min post-thaw (*p* < 0.05). Nevertheless, no effect was observed in PDO-treated PFE samples (*p* > 0.05). When PHL was added to GFE samples, MMP decreased in the presence of the highest concentration at 30 min post-thaw (*p* < 0.05), but this difference did not persist at 240 min post-thaw (*p* > 0.05). Concerning PFE samples, MMP decreased at 30 min post-thaw when PHL was added at final concentrations of 500 μmol/L and 1000 μmol/L (*p* < 0.05), and this effect only persisted at 240 min post-thaw in the presence of PDO at the highest concentration (*p* < 0.05).

#### 2.2.5. Intracellular Levels of Superoxides (O_2_^−^•)

Intracellular levels of superoxides were evaluated through a co-staining with hydroethidine and YO-PRO-1 in GFE and PFE samples in order to determine the effects of AQP inhibition on sperm function (Figure 6). Intracellular levels of superoxides increased post-thaw in the presence of PDO, regardless of its concentration in GFE (*p* < 0.05). Regarding PFE, the percentage of spermatozoa with high intracellular levels of O_2_^−^• increased in the presence of the highest concentration at 30 min post-thaw (*p* < 0.05), whereas at 240 min post-thaw this effect was observed in the presence of both 1 and 10 mmol/L PDO (*p* < 0.05). There were no differences between PHL-treated samples and the control in terms of O_2_^−^• levels in the PFE group (*p* > 0.05).

#### 2.2.6. Intracellular Levels of Hydrogen Peroxides (H_2_O_2_)

Intracellular levels of hydrogen peroxides were assessed through co-staining with 2′,7′-dichlorodihydrofluorescein diacetate (H_2_DCFDA) and PI fluorochromes in order to determine the effect of AQP inhibitors on this sperm functional parameter (Figure 7). When GFE samples were treated with PDO, intracellular levels of hydrogen peroxides were higher than in the control at both post-thaw time points (*p* < 0.05). In the PFE group, this effect was observed in the presence of both 1 and 10 mmol/L PDO at 30 min post-thaw (*p* < 0.05), whereas at 240 min post-thaw there were no differences between the control and any of the PDO treatments (*p* > 0.05). Regarding treatments with PHL, no variations in the intracellular levels of hydrogen peroxides were observed at any time point or concentration, either in GFE or in PFE group (*p* > 0.05).

## 3. Discussion

Despite several studies conducted in the last decade to identify the presence of AQPs in sperm of different mammalian species (reviewed in [18]), neither their precise function nor their mechanism of action have been fully addressed. In this context, sperm cryotolerance has been correlated with relative levels of AQP3 and AQP7 (which are both GLPs) [25]. Moreover, there is recent evidence suggesting that GLPs have a more relevant role than orthodox AQPs in the cryotolerance of boar and stallion spermatozoa [26,27]. Thus, this study has used different AQP inhibitors to unveil the functional relevance of AQPs during cryopreservation, comparing ejaculates with good (GFE) and poor (PFE) freezability. To this end, two inhibitors were added to the extender at three different concentrations: PDO at 0.1, 1 and 10 mmol/L, and PHL at 250, 500, and 1000 µmol/L. Whereas PDO inhibits orthodox AQPs (AQP1, AQP2, AQP5, and AQP4) with high efficiency, it is less efficient as a GLPs inhibitor (the family to which AQP3, AQP7, and AQP9 belong) is lower [28,29]. PHL, on the other hand, inhibits both AQP3 and AQP7 [32,33,34]. The effects of each inhibitor on sperm function and survival during cryopreservation were evaluated in terms of sperm motility, viability, membrane lipid disorder, MMP, and intracellular levels of ROS (reactive oxygen species). With regard to PDO, its presence in GFE samples increased total and progressive motility and sperm viability, decreased membrane lipid disorder, and increased MMP and ROS levels. In PFE samples, however, only the increase in ROS levels was observed. On the other hand, when PHL was added to extender, a decrease in TMOT, PMOT, sperm viability, and in the percentage of spermatozoa with low membrane lipid disorder and with high MMP was observed. In PFE, the effect on sperm viability was less evident than in GFE, the effect on MMP was more drastic and, whereas TMOT and membrane lipid disorder were transitionally lower at 30 min post-thaw, this difference with regard to the control was less apparent than in GFE and did not persist at 240 min post-thaw.

Differences between the effects of each AQP inhibitor are potentially caused by their different affinity for GLPs and the collateral effects on other sperm structures and molecules. In this context, PHL is able to freely permeate the plasma membrane due to its hydrophobicity [35], and it binds to GLPs through an internal binding site [36]. Therefore, GLP inhibition through PHL decreases the permeability to both water and other small solutes, which suggests that the detrimental effects observed might be a consequence of the impairment in this transport. The fact that sperm quality and function are less affected in PFE than in GFE, either because there is no effect in PFE or because that effect is less dramatic in PFE than in GFE, is in agreement with the different levels of GLPs (AQP3 and AQP7) between these groups, which, as described by Prieto-Martinez et al. (2017), are higher in samples with better cryotolerance. Moreover, the transitional decrease in total motility and in membrane lipid disorder at 30 min post-thaw in PFE could be masked at 240 min post-thaw because of the poor quality of these samples. Finally, the higher impairment of MMP in PFE than in GFE does not have an apparent cause, but it could be a consequence of collateral effects of PHL on other sperm proteins, such as SLC2A2 (solute carrier family 2, also known as GLUT2) [37]. Since this protein is involved in the uptake of monosaccharides, which are the main source of energy for sperm [38], this could be the reason of the lower MMP observed in the presence of PHL. Considering all the aforementioned, it can be suggested that the detrimental effect of GLP inhibition by PHL depends on the intrinsic freezability of the ejaculate.

Regarding PDO, it remains inside the pore of orthodox AQPs, whereas the broader pore of GLPs allows its free permeability [29]. One could expect the effect of GLP inhibition through PDO on sperm quality and function to be similar to the effect of PHL treatments, but our group recently showed that the inhibition of GLPs through PDO does not seem to be as efficient as through PHL, and that PDO is a potential cryoprotective agent [26]. Moreover, we showed that the inhibition of orthodox AQPs during cryopreservation does not seem to have detrimental effects on sperm quality and function after thaw and thus, the effects of PDO supplementation seems an exclusive consequence of the interactions with GLPs and of its cryoprotective effects on spermatozoa [26]. The present work evidenced that the effects of PDO treatments were more apparent in GFE than in PFE, as was also observed in the PHL treatments, and the consequences of PDO supplementation were consistent to the ones from previous works [26]. Concerning the GFE group, the addition of PDO seemed to better preserve the overall quality and function of sperm in terms of TMOT, PMOT, viability, and membrane lipid disorder, which might be a consequence of the expected cryoprotective effect. In this context, PDO has been used as a cryoprotective agent in cryopreservation protocols for canine ovarian cortices [39], and Widiasih et al. [40] demonstrated that PDO has milder detrimental effects on human sperm quality than glycerol. Moreover, even if the increase in MMP does not have an apparent cause, it is also a good sperm quality indicator and it might be the cause of the higher percentage of viable spermatozoa with high levels of ROS, since mitochondria are the main source of these molecules. Even if, to the best of our knowledge, the effects of PDO on oxidative stress have not been previously reported, AQP3 participates in H_2_O_2_ transport [41]. Therefore, PDO inhibition of H_2_O_2_-efflux might contribute to the increase in H_2_O_2_ intracellular concentration, which has a relevant role in mammalian sperm function [10]. In fact, ROS play a crucial role in the activation of the molecular pathways that are associated with sperm capacitation but, when generated in excess, oxidative stress can alter the maintenance of sperm function through both the induction of lipid peroxidation and DNA damage (reviewed in [42]). While the increase in ROS, which can be considered to have a negative effect on sperm quality, was more evident in GFE than in PFE, the positive effects on sperm quality and function were only detected in the GFE group. Therefore, it seems that both positive and negative effects of PDO rely upon the intrinsic ejaculate freezability.

## 4. Materials and Methods

### 4.1. Boars and Ejaculates

In this study, 42 ejaculates from separate Piétrain boars (*n* = 27) were used. Boars (aged between 15 and 24 months old) were housed with controlled climatic conditions and were fed a standard diet in a local farm (Semen Cardona, Cardona, Barcelona, Spain). Manual collection of sperm-rich fractions was performed twice a week with an interval of three days between collections. Samples were subsequently diluted 1:1 (v/v) in a commercial semen extender (Vitasem LD; Magapor S.L., Zaragoza, Spain), and then packaged in bags and stored at 17 °C. Within 5 h post-extraction, samples were transported to the laboratory. Ejaculates were then pooled in pairs (*n* = 21) and were subsequently divided into two different fractions. The first fraction was used to assess sperm quality parameters in fresh samples. The second one was divided into seven different sub-fractions, one of which was the control and the remaining six were treated with three different concentrations of two AQP inhibitors and subsequently cryopreserved. Sperm quality evaluation was also performed in cryopreserved samples after thawing.

### 4.2. AQP Inhibitors

Two AQP inhibitors, 1,3-propanediol (PDO, Sigma-Aldrich, St. Louis, MO, USA) and phloretin, (PHL, Sigma-Aldrich) were added to the freezing medium before sample cryopreservation. For each inhibitor, a stock solution was prepared; whereas PDO was diluted at 100 mmol/L in cryopreservation medium (LEYGO medium, composition detailed in the following section), PHL was diluted at 365 mmol/L in methanol (Fisher Chemical, ThermoFisher Scientific; Waltham, MA, USA). Samples were supplemented with three different concentrations of each inhibitor: 0.1, 1, and 10 mmol/L of PDO; and 250, 500, and 1000 μmol/L of PHL. Preliminary experiments were conducted to determine the minimum concentration of each inhibitor at which an effect was observed, and the maximum concentration at which cytotoxic effects appeared.

Concerning PHL-treated samples, concentrations of methanol to which spermatozoa were exposed were lower than 0.5% (v/v). We confirmed that, at these concentrations, sperm quality parameters evaluated were not altered (data not shown).

### 4.3. Boar Sperm Cryopreservation

Samples were cryopreserved to determine the effects of AQP inhibition on the cryotolerance of ejaculates. Fifty-mL aliquots of the fraction of each ejaculate pool that was intended for cryopreservation were centrifuged at 2400× *g* for 3 min at 15 °C. After discarding supernatants, β-lactose-egg yolk freezing medium (LEY), which consisted of 80% (v/v) lactose (Sigma-Aldrich) and 20% (v/v) egg yolk, was used to resuspend pellets at a final concentration of 1.5 × 10^9^ spermatozoa/mL. Sample cooling was performed in a programmable, controlled-rate freezer (Icecube14S-B; Minitüb Ibérica SL; Tarragona, Spain), with a cooling rate of −0.1 °C/min (180 min) and a final temperature of 5 °C. At this point, LEYGO medium, consisting of LEY medium supplemented with 6% (v/v) glycerol (Sigma-Aldrich) and 1.5% Orvus ES Paste (Equex STM; Nova Chemical Sales Inc., Scituate, MA, USA), was used to dilute samples to a final concentration of 1 × 10^9^ spermatozoa/mL. Seven sub-fractions were subsequently obtained from this fraction: a non-treated control and one for each concentration and inhibitor. Sample packaging was carried out in 0.5 mL plastic straws (Minitüb Ibérica, S.L.); filled straws were placed in a programmable, controlled-rate freezer and exposed to the following cooling rates [20]: −6 °C/min from 5 to −5 °C (100 s); −39.82 °C/min from −5 to −80 °C (113 s); hold at −80 °C for 30 s; and cooled at −60 °C/min from −80 to −150 °C (70 s). Samples were then plunged and stored in liquid nitrogen (−196 °C).

Sample thawing was performed in order to evaluate sperm quality parameters in cryopreserved samples. Two straws per treatment from each pool were immersed in a water bath at 38 °C for 15 s and subsequently diluted 1:3 (v/v) in pre-warmed (38 °C) Beltsville Thawing Solution (BTS) [27]. Sperm samples were then incubated at 38 °C for 240 min, and sperm quality was evaluated at two different time points: 30 min and 240 min. These time points are consistent with the protocols from Vilagran et al. [24] and Prieto-Martínez et al. [25] for the evaluation of sperm function and survival in cryopreserved boar semen. The evaluation at 30 min post-thaw allows the stabilization of spermatozoa after both thermal and osmotic stresses that occur upon thawing, but it is also at this time point that the short-term detrimental effects of cryopreservation are evident. Regarding the analysis at 240 min post-thaw, it was set to assess the quality and function of frozen-thawed spermatozoa within the insemination-to-ovulation interval recommended for cryopreserved doses [43]. In addition, this time period is coincident with the time that boar spermatozoa need to capacitate, and therefore, it corresponds to the time at which capacitation-like changes are apparent [44]. Therefore, the evaluation of sperm function and survival at 240 min post-thaw is performed in order to ensure that the insemination-to-ovulation interval recommended for cryopreserved boar semen is covered, and to assess capacitation-like changes, which correspond to the long-term detrimental effects of cryopreservation.

### 4.4. Sperm Motility

Sperm motility evaluation was performed with a computer-assisted sperm analysis (CASA) system, consisting of a negative phase-contrast microscope (Olympus BX41; Olympus, Tokyo, Japan), a video camera and ISAS software (Integrated Sperm Analysis System V1.0; Proiser SL, Valencia, Spain), in both fresh (extended) and frozen-thawed samples. Before sperm motility assessment, extended samples required 15 min of incubation at 38 °C, whereas frozen-thawed samples were directly examined after their respective incubation times at 38 °C: 30 min or 240 min. Once incubated, 5 µL of each sample was examined in a pre-warmed (38 °C) Makler counting chamber (Sefi-Medical Instruments, Haifa, Israel), under a negative phase-contrast field (Olympus 10× 0.30 PLAN objective; Olympus). Three replicates of at least 1000 spermatozoa were examined for each treatment.

The following parameters were recorded in each motility assessment: TMOT (%) and PMOT (%); curvilinear velocity (VCL, μm/s); straight line velocity (VSL, μm/s); average path velocity (VAP, μm/s); amplitude of lateral head displacement (ALH, μm); beat cross frequency (BCF, Hz); linearity (LIN, %), which was calculated as LIN = VSL/VCL×100; straightness (STR, %), resulting from VSL/VAP×100; and motility parameter wobble (WOB, %), obtained from VAP/VCL×100 [45]. Spermatozoa considered as motile were those presenting a VAP value equal to or higher than 10 μm/s; progressively motile spermatozoa presented an STR value equal to or higher than 45% [46]. The corresponding mean ± standard error of the mean (SEM) was calculated for each parameter.

### 4.5. Flow Cytometry

Flow cytometry was used to evaluate five different sperm quality and functionality parameters in both fresh and frozen-thawed samples: sperm viability, membrane lipid disorder, MMP, intracellular levels of superoxides and intracellular levels of peroxides. All fluorochromes were purchased from ThermoFisher Scientific (Waltham, MA, USA). Prior to the addition of the corresponding fluorochromes, samples were diluted to a final concentration of 1 × 10^6^ spermatozoa/mL. Once stained, samples were incubated at 38 °C. A total of three replicates per sample were assessed for each parameter.

Samples were analyzed in a Cell Lab Quanta^TM^ SC cytometer (Beckman Coulter; Fullerton, CA, USA). Samples were excited at a power of 22 mW through an argon ion laser (488 nm). The Coulter principle, which measures changes of electrical resistance in an electrolyte solution by suspended, non-conductive particles, allowed cell diameter/volume assessment. In this system, forward scatter (FS) is replaced by electronic volume (EV). For EV-channel calibration, 10-μm Flow-Check fluorospheres (Beckman Coulter) were positioned at channel 200 on the EV-scale.

Three optical filters were used for fluorescence detection: FL1 (Dichroic/Splitter, DRLP: 550 nm, BP filter: 525 nm, detection width: 505–545 nm), FL2 (DRLP: 600 nm, BP filter: 575 nm, detection width: 560–590 nm), and FL3 (LP filter: 670 nm/730 nm, detection width: 655–685 nm). FL1 detected green fluorescence from SYBR14, YO-PRO-1, JC-1 monomers (JC-1_mon_), and 2′,7′-dichlorofluorescein (DCF^+^); FL2 detected orange fluorescence from JC-1 aggregates (JC-1_agg_); and FL3 was used to detect red fluorescence from PI, M540, and ethidium (E^+^). The signal was logarithmically amplified, and particular staining methods were considered for photomultiplier setting.

EV and side scatter (SS) were measured and linearly recorded for all particles, and the sheath flow rate was set at 4.17 µL/min. On the EV channel, the analyzer threshold was adjusted to exclude subcellular debris (particle diameter <7 μm) and cell aggregates (particle diameter >12 μm). Therefore, on the basis of EV/SS distributions, the sperm-specific events were positively gated. A minimum of 10,000 events were evaluated per replicate, and three replicates were analyzed per sample.

Flowing Software (Ver. 2.5.1; University of Turku, Finland) was used for data analysis, according to the recommendations of the International Society for Advancement of Cytometry (ISAC). Following Petrunkina et al. [47], the percentage of non-sperm debris particles of the SYBR14^−^/PI^−^ population was used to correct the events corresponding to double-negative particles of all protocols. For each parameter, the corresponding mean ± SEM was calculated.

#### 4.5.1. Sperm Viability

Sperm viability was evaluated through the LIVE/DEAD sperm viability kit (Molecular Probes, Eugene, OR, USA) following the protocol of Garner and Johnson [48]. Briefly, sperm were incubated with a final concentration of 100 nmol/L SYBR14 for 10 min, and with PI at a final concentration of 12 µmol/L for 5 min. Flow cytometry dot-plots showed three different sperm populations: (1) viable, green-stained spermatozoa (SYBR14^+^/PI^−^); (2) non-viable, red-stained spermatozoa (SYBR14^−^/PI^+^); (3) non-viable spermatozoa stained in both green and red (SYBR14^+^/PI^+^). The unstained, non-sperm particles (SYBR14^−^/PI^−^) were not included for the calculation of the final percentages of the three aforementioned populations. SYBR14 fluorescence spill over into FL3 channel was compensated (2.45%).

#### 4.5.2. Sperm Membrane Lipid Disorder

Samples were co-stained with M540 and YO-PRO-1 to evaluate sperm membrane lipid disorder, according to the protocol from Rathi et al. [49] with minor modifications [50]. Decreases in packing order of phospholipids from the outer monolayer of the plasma membrane are detected by M540, which is the basis of this protocol. M540 was added to samples at a final concentration of 2.6 µmol/L, and YO-PRO-1 at a final concentration of 25 nmol/L, prior to 10 min of incubation. Four different populations were identified in flow cytometry dot plots: (1) viable spermatozoa presenting low membrane lipid disorder (M540^−^/YO-PRO-1^−^); (2) viable spermatozoa presenting high membrane lipid disorder (M540^+^/YO-PRO-1^−^); (3) non-viable spermatozoa presenting low membrane lipid disorder (M540^−^/YO-PRO-1^+^); and (4) non-viable spermatozoa presenting high membrane lipid disorder (M540^+^/YO-PRO-1^+^). The percentage of SYBR14^−^/PI^−^ particles corresponding to non-sperm debris particles was used to correct the percentages of viable spermatozoa presenting low membrane lipid disorder (M540^−^/YO-PRO-1^−^), the percentages of the other populations were recalculated. Data were not compensated.

#### 4.5.3. Mitochondrial Membrane Potential (MMP)

Samples were stained with JC1 following the protocol of Ortega-Ferrusola et al. [51] with minor modifications in order to evaluate MMP. JC1 molecules (5,5′,6,6′-tetrachloro-1,1′,3,3′tetraethyl-benzimidazolylcarbocyanine iodide) remain as monomers (JC1_mon_) in the presence of low MMP, whereas, in the presence of high MMP, they form aggregates (JC1_agg_). Briefly, samples were incubated with JC1 at a final concentration of 0.3 µmol/L for 30 min. Three different populations were observed in flow cytometry dot-plots: (1) low MMP-spermatozoa (JC1_mon_; FL1^+^/FL2^−^); (2) high MMP-spermatozoa (JC1_agg_; FL1^−^/FL2^+^); and (3) spermatozoa with heterogeneous mitochondria in the same cell (JC1_agg_ and JC1_mon_; FL1^+^/FL2^+^). The percentage of SYBR14^−^/PI^−^ particles was used to correct the percentages of double-negative particles (FL1^−^/FL2^−^), and the percentages of the other three populations were recalculated. The sum of populations (2) and (3) corresponded to high MMP-spermatozoa. FL1 spill-over into the FL2 channel was compensated (51.70%).

#### 4.5.4. Intracellular Levels of Superoxides (O_2_^−^•)

Samples were co-stained with HE and YO-PRO-1 in order to evaluate the levels of intracellular superoxides (O_2_^−^•), following the protocol from Guthrie & Welch [52]. HE is oxidized at an intracellular level by O_2_^−^• radicals to E^+^ and other products. In brief, samples were incubated with HE at a final concentration of 4 µmol/L and YO-PRO-1 at a final concentration of 40 nmol/L for 20 min. Flow cytometry dot-plots allowed the identification of four different sperm populations: (1) viable spermatozoa with low O_2_^−^•levels (E^−^/YO-PRO-1^−^); (2) viable spermatozoa with high O_2_^−^•levels (E^+^/YO-PRO-1^−^); (3) non-viable spermatozoa with low O_2_^−^• levels (E^−^/YO-PRO-1^+^); and (4) non-viable spermatozoa with high O_2_^−^• levels (E^+^/YO-PRO-1^+^). The proportions of non-sperm debris particles (SYBR14^−^/PI^−^ particles) were used to correct the percentages of viable spermatozoa with low O_2_^−^•levels (E^−^/YO-PRO-1^−^). YO-PRO-1 spill over into the FL3-channel was compensated (5.06%).

#### 4.5.5. Intracellular Levels of Hydrogen Peroxide (H_2_O_2_)

Spermatozoa were co-stained with H_2_DCFDA and PI following the protocol set by Guthrie & Welch [52] with minor modifications to assess intracellular levels of hydrogen peroxide (H_2_O_2_). The non-fluorescent probe H_2_DCFDA is de-esterified and oxidized at an intracellular level into highly fluorescent DCF^+^. Briefly, samples were incubated with H_2_DCFDA at a final concentration of 200 µmol/L and PI at a final concentration of 12 µmol/L for 30 min. Four different sperm populations were identified in flow cytometry dot-plots: (1) viable spermatozoa with low levels of peroxides (DCF^−^/PI^−^); (2) viable spermatozoa with high levels of peroxides (DCF^+^/PI^−^); (3) non-viable spermatozoa with low levels of peroxides (DCF^−^/PI^+^); and (4) non-viable spermatozoa with high levels of peroxides (DCF^+^/PI^+^). The proportions of non-sperm debris particles from the SYBR14^−^/PI^−^ quadrant were used to correct the percentages of live spermatozoa with low levels of peroxides (DCF^−^/PI^−^); the percentages of the other populations were recalculated. DCF-spill over into FL3-channel was compensated (2.45%).

### 4.6. Statistical Analyses

A statistical package (IBM SPSS Statistics 25.0; Armonk, New York, USA) was used to analyze the results obtained in this work. Shapiro–Wilk and Levene tests were run to assess the distribution of data and homogeneity of variances, respectively. Thereafter, ejaculate pools were classified as good (GFE) or poor (PFE) freezability based on their total and progressive sperm motilities, and sperm viability (% SYBR14^+^/PI^−^ spermatozoa) at 30 min post-thaw through a two-step hierarchical cluster analysis using the log-likelihood distance and the Bayesian–Schwarz criterion. For each sperm parameter, a mixed model was subsequently run. The cryopreservation step was the intra-subjects factor (i.e., fresh, frozen-thawed at 30 min, frozen-thawed at 240 min), the treatment at a given concentration (C, PDO, or PHL) and the ejaculate group (GFE or PFE) were the fixed-effects factors (inter-subject), and the ejaculate pool was the random-effects factor. Pair-wise comparisons were tested through a post-hoc Sidak test, and the level of significance was set at *p* ≤ 0.05. Data are shown as mean ± standard error of the mean (SEM).

## 5. Conclusions

In conclusion, the inhibition of AQPs through two different inhibitors has more detrimental effects on ejaculates with good cryotolerance than in those with poor cryotolerance. Moreover, the improvement of post-thaw sperm quality and functionality with PDO as a supplementary CPA is exclusively evident in GFE.

## Abbreviations

AQP	Aquaporin
ALH	Amplitude of lateral head displacement
BCF	Beat cross frequency
BTS	Beltsville Thawing Solution
CASA	Computer-assisted sperm analysis
CPA	Cryoprotective agent
DCF^+^	2′,7′-dichlorofluorescein
E^+^	Ethidium
EV	Electronic volume
FS	Forward scatter
GFE	Good freezability ejaculates
GLP	Aquaglyceroporins
H_2_DCFDA	2′,7′-dichlorodihydrofluorescein diacetate
H_2_O_2_	Hydrogen peroxide
HE	Hydroethidine
ISAC	International Society for Advancement of Cytometry
JC-1	5,5′,6,6′-tetrachloro-1,1′,3,3′tetraethyl-benzimidazolylcarbocyanine iodide
JC-1_agg_	JC-1 aggregates
JC-1_mon_	JC-1 monomers
LEY	β-Lactose–egg yolk–glycerol
LEYGO	β-Lactose–egg yolk–glycerol–Orvus ES Paste
LIN	Linearity
M540	Merocyanine 540
MMP	Mitochondrial membrane potential
O_2_^−^•	Superoxide
PDO	1,3-propanediol
PfAQP	Plasmodium falciparum aquaporin
PFE	Poor freezability ejaculates
PHL	Phloretin
PI	Propidium iodide
PMOT	Progressive motility
ROS	Reactive oxygen species
SEM	Standard error of the mean
SLC2A2	Solute carrier family 2, facilitated glucose transporter member 2
SS	Side scatter
STR	Straightness
TMOT	Total motility
VAP	Average path velocity
VCL	Curvilinear velocity
VSL	Straight line velocity
WOB	Motility parameter wobble
PMOT	Progressive motility
ROS	Reactive oxygen species
SEM	Standard error of the mean
SLC2A2	Solute carrier family 2, facilitated glucose transporter member 2
SS	Side scatter
STR	Straightness
